# Behavioral assays reveal mechanisms of supercolony formation in odorous house ants

**DOI:** 10.1038/s41598-023-35654-y

**Published:** 2023-06-02

**Authors:** Grzegorz Buczkowski, Sihui Wang, Bruce A. Craig

**Affiliations:** 1 Department of Entomology, Purdue University, West Lafayette, IN 47907 USA; 2 Department of Statistics, Purdue University, West Lafayette, IN 47907 USA

**Keywords:** Invasive species, Behavioural ecology, Biodiversity, Community ecology, Conservation biology, Invasive species, Urban ecology

## Abstract

The formation of expansive multi-nest and multi-queen supercolonies is perhaps the most important factor responsible for the ecological success of invasive ants. The odorous house ant, *Tapinoma sessile*, is a widespread ant native to North America. *T. sessile* is a challenging urban pest, but also serves as an interesting system to study ant social organization and invasion biology. This is due to its remarkable dichotomy in colony social and spatial structure between natural and urban environments. Natural colonies typically consist of a small number of workers, inhabit a single nest, and are monogyne whereas urban colonies show extreme polygyny and polydomy and form large supercolonies. The current study examined the extent to which *T. sessile* colonies from different habitats (natural vs. urban) and social structures (monogynous vs. polygynous) exhibit aggression toward alien conspecifics. Additionally, interactions between mutually aggressive colonies were examined in colony fusion experiments to assess the potential role of colony fusion as a mechanism leading to supercolony formation. Aggression assays demonstrated high levels of aggression in pairings involving workers from different urban colonies and workers from different natural colonies, but low aggression in pairings involving queens from different urban colonies. Colony merging tests demonstrated that urban *T. sessile* colonies are highly aggressive to each other, but capable of fusing under laboratory conditions when competing for limited nesting and food resources. Despite highly aggressive interactions and relatively high worker and queen mortality, all colony pairs merged in 3–5 days. Fusion occurred after most workers died and the survivors merged. This result suggests that the success of *T. sessile* in urban areas may be driven, at least in part, by successful colony mergers of unrelated colonies which may be determined by ecological constraints such as seasonal shortages in nest and/or food availability. In summary, two independent factors including the growth of a single colony and/or the merger of multiple colonies may be responsible for the evolution of supercolonies in invasive ants. Both processes may be happening simultaneously and may act synergistically to produce supercolonies.

## Introduction

One of the central questions in invasion biology concerns the invasiveness of species and the invasibility of habitats. Which species are most likely to become invasive? Which habitats are most susceptible to invasion? Despite decades of research and management, it has proven difficult to identify traits that consistently predict invasiveness^1,2^. The proportion of introduced species that ultimately become invasive is estimated to be lower than 1 percent^3^ and a perplexing question in invasion ecology is why certain species become invasive while others do not. Invasion success may depend on pre-adaptations present in the native range, characters evolving de novo after introduction, and various interactions between these two factors.

Invasive ants are highly prolific invaders and among the world’s most damaging pests^4,5^. Invasive ants commonly share a suite of traits including multiqueen colony structure, extensive polydomy, and success in anthropogenic habitats. Many invasive ant species form supercolonies, extremely large colonies consisting of millions of individuals over large geographic areas (e.g. *Linepithema humile*^6^; *Pheidole megacephala*^7^; *Anoplolepis gracilipes*^8^; *Nylanderia fulva*^9^). Supercolonial populations are characterized by the absence of territorial behavior and intraspecific aggression, dense network of interconnected nests genetically indistinguishable from each other, high population densities, colony dispersal by budding, and ecological dominance over native organisms^10^. Additionally, many supercolonial ants are pests in agricultural, urban, and wildland habitats^11^.

The odorous house ant, *Tapinoma sessile* (Say, 1836) is considered one of the most adaptive and widespread ant species found in North America and is a common nuisance pest^12^. In addition to its pest status, *T. sessile* is of interest as a model system for understanding the factors that lead to variable queen number and nesting strategy across ant species, as well as possible insights into the evolution of invasiveness in ants. In natural environments, *T. sessile* colonies are small and consist of a few hundred workers and one or a few queens^13–15^. However, in urban environments, colonies tend to act as an invasive species and consist of large supercolonies with millions of workers and thousands of queens spread across multiple, interconnected nesting sites^16^. Facultative polygyny in *T. sessile* serves as an interesting system for the study of ant social organization and invasion biology. Similar to other invasive ants, urban populations of *T. sessile* are correlated with reduced abundance and diversity of other ant species^17,18^. Recently, a non-native, supercolonial population of *T. sessile* was discovered in Hawaii, a rare example of a temperate-origin ant invading a tropical region^19^. Therefore, *T. sessile* provide an interesting example of a native “invasive” ant and serve as a model for understanding biological invasions.

This study examined the extent to which *T. sessile* colonies from different habitats (natural vs. urban) and social structures (monogynous vs. polygynous) exhibit aggression toward alien conspecifics. The overall objective for the study was to examine potential behavioral mechanisms that may lead supercolony formation in *T. sessile*. The definition of supercolony is not well characterized and remains controversial. However, for the purpose of this study, we define supercolonies as large, multi-nest, multi-queen urban colonies and contrast them with significantly smaller, single-nest, single-queen natural co1onies^13,15^. Previous studies have examined behavioral interactions in *T. sessile* but focused on aggression within individual colonies to confirm the existence of supercolonies in the native^17^ and introduced range^19^. Additionally, Blumenfeld et al.^15^ performed a large-scale molecular, chemical, and behavioral analysis of *T. sessile* in natural and urban habitats and demonstrated high aggression among nests across habitats suggesting little correlation between aggression and geographic distance and genetic or chemical differentiation. The goals for the current study were four-fold. The first objective examined aggression levels in worker ants collected from different colonies within each habitat type (urban vs. natural). In many species of ants, polygyny is associated with reduced aggression with unicolonial populations as the most extreme example^10^. The hypothesis was that *T. sessile* colonies collected in urban areas would show significantly lower aggression levels towards each other relative to colonies collected in natural areas. This hypothesis was previously tested in Blumenfeld et al.^15^ and found to be true in 1 of 3 populations. The second objective compared aggression levels between workers and queens collected from different colonies in urban areas. In ants, aggression assays are almost exclusively performed on workers^20^ or colony fragments containing workers and queens^21^. Aggression assays utilizing queens are rarely if ever performed despite the importance of queens for colony founding and survival. The hypothesis was that queens would be significantly less aggressive (and less aggressed) relative to workers due to caste-specific differences in tasks and behaviors. The third objective assessed aggression levels between queens from different colonies in urban areas. The hypothesis was that in the absence of workers aggression between queens would be low which could lead to primary polygyny through pleometrotic foundation. The final objective was to determine if unrelated urban colonies of *T. sessile* are capable of fusing under laboratory conditions. The hypothesis was that in the absence of natural barriers preventing their encounters colonies would permanently fuse leading to the formation of mixed, numerically superior colonies. Blumenfeld et al.^15^ demonstrated that workers in urban colonies of *T. sessile* have high relatedness (r = 0.65) which suggests that merging of genetically different colonies is unlikely to occur in field situations. However, Vasquez and Silverman^21^ investigated colony fusion in *L. humile* and demonstrated that mutually aggressive colonies with dissimilar genetic and cuticular hydrocarbon profiles fused in the absence of barriers preventing their encounters. The final objective tested whether a similar process may be happening in *T. sessile*.

## Results

**Figure 1:**
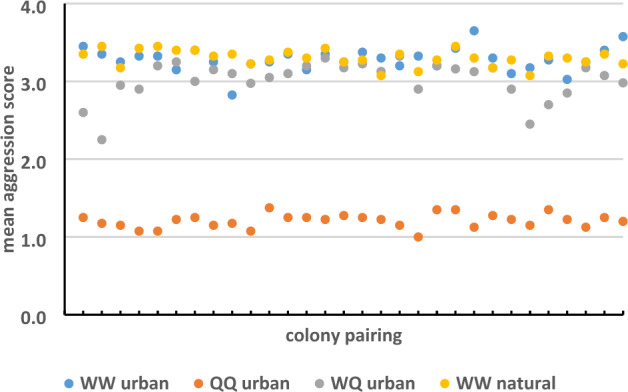
Mean aggression scores (± stdev excluded for clarity) in *T. sessile* colonies.

Aggression assays demonstrated high levels of aggression among workers from urban colonies and workers from natural colonies (Fig. 1). The mean aggression score in WW pairings from urban colonies was 3.28 ± 0.16 and the maximum aggression score (level 4) was recorded at least once in all replicates of all colony pairings. The aggression score of “1” was recorded in 4% of interactions, “2” in 10% of interactions, “3” in 41% of interactions, and “4” in 45% of interactions. In total, non-injurious aggression accounted for 14% of interactions, while injurious aggression accounted for 86% of interactions. There was no difference in mean aggression scores among urban WW pairings (log likelihood = 0.5, *P*-value = 0.48).

A similar trend was recorded in WW pairings from natural colonies. The mean aggression score was 3.30 ± 0.10 and the maximum aggression score (level 4) was recorded at least once in all replicates of all colony pairings. The aggression score of “1” was recorded in 2% of interactions, “2” in 19% of interactions, “3” in 25% of interactions, and “4” in 54% of interactions. In total, non-injurious aggression accounted for 21% of interactions, while injurious aggression accounted for 79% of interactions. There was no difference in mean aggression scores among natural WW pairings (log likelihood = 0.7, *P*-value = 0.53).

Additionally, there was no significant difference in mean aggression scores obtained for urban WW pairings versus natural WW pairings (*P*-value = 0.98, df = 46.02).

Aggression assays also demonstrated high levels of aggression between workers and queens collected in urban locations (WQ interactions, Fig. 1). The mean aggression score in urban WQ pairings was 3.06 ± 0.18 and the maximum aggression score (level 4) was recorded in 111 out of 120 (93%) replicate tests. The aggression score of “1” was recorded in 5% of interactions, “2” in 13% of interactions, “3” in 53% of interactions, and “4” in 29% of interactions. In total, non-injurious aggression accounted for 18% of interactions, while injurious aggression accounted for 82% of interactions. There was no difference in mean aggression scores among urban WQ pairings (log likelihood = 0.7, *P*-value = 0.53). Behavioral observations indicate that in WQ interactions, workers always initiated aggression towards queens. Queens never attacked workers and generally attempted to run away, but sometimes fought back when seized by the worker and unable to escape. In contrast to urban WW pairings and urban WQ pairings, aggression was absent in urban QQ pairings (Fig. 1). The mean aggression score was 1.21 ± 0.09. The aggression score of “1” was recorded in 79% of interactions and “2” in 21% of interactions. In total, non-injurious aggression accounted for 100% of interactions, and injurious aggression was never observed. There was no difference in mean aggression scores among urban QQ pairings (log likelihood = 0.7, *P*-value = 0.40).

**Figure 2:**
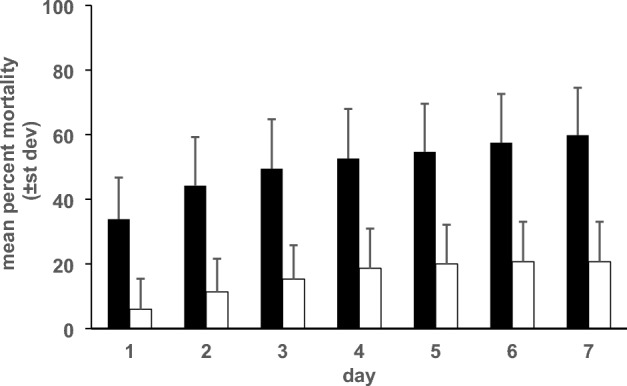
Mean percent mortality (± stdev) in *T. sessile* workers (closed bars) and queens (open bars) in colony merging experiments.

Colony fusion varied across colony pairs and the percentage of fused colonies increased over time (Table 1). Results demonstrate that all colony pairs were highly aggressive and all pairs experienced relatively high worker and queen mortality (Fig. 2). On day 7, mean mortality was 61 ± 15% in the workers and 18 ± 12% in the queens. Behavioral observations demonstrate that the majority of workers that died during the 7 day testing period died within the first 24 h as the colonies explored the arena and competed for the central nest. Fusion occurred within 24 h in 8 out of 18 (44%) replicates. Fusion generally occurred when worker mortality was either relatively low and most workers accepted each other or when worker mortality was relatively high and most workers died and the survivors merged. Despite highly aggressive interactions and high mortality, all colony pairs merged in 3–5 days. Paint markings on the queens in all merged replicates revealed that queens of both colonies resided in the central nest. Brood and workers were present as well and behavioral re-introduction tests demonstrated the survival of workers from both colonies. However, the relative survival of workers from both colonies is unknown and it is possible that the colonies experienced unequal mortality or that they remained spatially partitioned within the nest.

**Table 1:** Initial level of worker-worker aggression in dyad interactions (mean ± stdev) and results of colony merging tests (out of 3 replicates).

Colony pair	Mean aggression score (N = 4)	Number of merged replicates						
		Day 1	Day 2	Day 3	Day 4	Day 5	Day 5	Day 7
1–2	3.5 ± 0.6	2	3	3	3	3	3	3
1–3	3.3 ± 0.8	1	1	2	2	3	3	3
2–5	3.1 ± 0.8	1	1	2	3	3	3	3
4–3	3.9 ± 0.4	1	1	1	2	3	3	3
6–4	3.3 ± 0.7	1	1	2	3	3	3	3
6–5	3.4 ± 0.5	2	2	2	2	3	3	3
1–1	1.1 ± 0.3	2	3	3	3	3	3	3
2–2	1.2 ± 0.2	3	3	3	3	3	3	3
3–3	1.1 ± 0.2	1	2	3	3	3	3	3
4–4	1.2 ± 0.2	3	3	3	3	3	3	3
5–5	1.0 ± 0.0	3	3	3	3	3	3	3
6–6	1.0 ± 0.0	1	3	3	3	3	3	3

Last 6 rows represent control tests (colony fragments belonging to the same colony).

## Discussion

Results demonstrate that *T. sessile* colonies are highly aggressive to each other, but capable of fusing under laboratory conditions when competing for limited nesting and food resources. Fusion of unrelated colonies may be one potential route by which *T. sessile* achieve supercolony size in urban areas. The fusion experiment used relatively small colonies in a restricted space and limited nest and food access and ecological constrains are known to shape the social and spatial organization of ant colonies. Nest site availability was the main driver regulating colony spatial and social structure in a cavity-nesting ant, *Leptothorax nylanderi*^22^. Seasonal overabundance of empty nests facilitated the fragmentation of larger colonies into smaller buds, while a shortage of nests led to the fusion of established, unrelated colonies. The colonies permanently merged after initial fighting and typically only one queen survived after fusion^22^. It’s unclear if a similar process happens in *T. sessile* and whether colonies in natural populations merge since approximately 60% of the workers forced to compete died. Additionally, Blumenfled et al.^15^ showed that colonies in urban areas have high levels of relatedness among workers suggesting it is highly unlikely that urban colonies could be the product of merging among different and presumably genetically distinct colonies. Current results and previous work on the origin of unicolonial ants^10,15^ support the hypothesis that large polydomous/polygynous colonies arise through the growth of a single colony, probably through the retention of daughter queens and dependent colony foundation.

Despite being a native species to North America, the success of *T. sessile* in urban habitats mirrors exotic invasive species in a variety of ways, from the shift towards large-scale polydomy and polygyny to reduced biodiversity^13–15,18^. Menke et al.^14^ examined several populations of *T. sessile* from across North America and found that large polygynous colonies of *T. sessile* were consistently more related to the local monogynous colonies than they were to more geographically distant polygynous colonies. This strongly implies that *T. sessile* is naturally pre-adapted for urban invasion and colony expansion in anthropogenic habitats and has successfully established polygynous colonies on several independent occasions, rather than a single switch to polygyny which was then spread by human-vectored dispersal. Blumenfeld et al.^15^ examined the phylogenetic, chemical, and behavioral traits of the four *T. sessile* clades described by Menke et al.^14^ and demonstrated that urbanization is an intense driver of evolution in *T. sessile* and that colony social structure is a plastic trait. Urban colonies were characterized by high levels of relatedness suggesting that queens are retained during colony growth and subsequent inheritance of daughter queens from the same linage.

In ants, secondary polygyny occurs when originally monogynous colony becomes polygynous later in its life cycle, generally as a result of accepting daughter queens of currently present queen into the colony and/or colony fusion^21,23^. One potential pathway for the evolution of supercolonies is the merger of multiple smaller colonies occupying a certain area to form a single supercolony. The merger could be between two monogynous colonies to form a polygynous colony or between two or more polygynous colonies to form a much larger and competitively superior polygynous colony. Previous research has shown that non-aggressive *L. humile* colonies attained greater productivity when merged^24^. The alternative pathway is that supercolonies develop over time from smaller colonies as colonies grow and spread to new areas via budding. Research to date suggests that colony mergers generally do not occur and that supercolonies develop over time from smaller colonies under favorable environmental conditions^14^. In ants, factors including territoriality, well-established foraging ranges, natural and artificial barriers, and sometimes large geographic distance between colonies may limit opportunities for mixing of different colonies. However, other factors including reproduction by budding, extreme vagility, constant internest exchange of individuals, movement of propagules by humans may promote opportunities for mixing and potential mergers. Our results suggest that successful colony mergers may occur under laboratory conditions, but they are unlikely to occur in natural populations given the high levels of relatedness within urban colonies, including highly polygynous ones^14^.

Urban colonies of *T. sessile* on mainland USA and introduced colonies in Hawaii are in many ways similar to introduced colonies of all other supercolonial invasive ant species. They often exhibit extreme polygyny and polydomy, form large supercolonies, and become dominant pests. One proposed mechanism for the formation of supercolonies in invasive ants is a genetic bottleneck which leads to the loss of genetic diversity and results in a loss of recognition cue diversity which ultimately leads to reduction in intraspecific aggression (e.g. *Linepithema humile*^25^). An alternative mechanism that may be happening concurrently is colony fusion where nearby competing colonies fuse to create larger and more productive colonies if the benefits from increased colony size supersede the costs of mortality sustained during merging. Colony fusion has been suggested as a route towards polygyny and unicoloniality in ants^26,27^. Vasquez and Silverman^21^ investigated colony fusion in *L. humile* and demonstrated that mutually aggressive colonies fused in the absence of barriers preventing their encounters. Colony fusion ranged from complete elimination to fusion and was regulated by the initial level of aggression between colonies as well as genetic and cuticular hydrocarbon similarity between interacting colonies whereby colonies with greater similarity were more likely to merge. A similar process may be happening in *T. sessile*. However, based on results by Blumenfeld et al.^15^, colony mergers due to genetic and/or cuticular hydrocarbon similarity are unlikely to occur in wild (non-laboratory) colonies of *T. sessile*. Phylogenetic and chemical analyses performed by Blumenfeld et al.^15^ on natural and urban *T. sessile* colonies uncovered clear differentiation among geographic localities and between natural and urban populations suggesting that mergers are unlikely to occur in field situations.

Aggression assays between workers and queens revealed high levels of aggression with injurious aggression in 82% of interactions. Workers immediately attacked foreign queens and behavioral observations indicate that aggression was entirely one-sided, where workers attacked queens and queens attempted to flee. This suggests that established colonies are unlikely to accept foreign queens that may accidentally stray into the territory of established colonies during budding events, mating flights, or unintentional transport by humans. In contrast to high aggression in worker-worker and worker-queen interactions, aggression was completely absent in queen-queen interactions. It is plausible that colony mergers may occur in situations where high worker aggression leads to the elimination of most aggressive worker phenotypes and the selective survival of queens, increasing the chance of fusion. However, it is unclear if workers produced in colonies resulting from fusion of unrelated queens would display homogenized recognition cues and non-aggressive behavior or whether they would be aggressive towards each other.

Aggression assays in ants are almost exclusively performed on worker ants^20,28^. This is because workers leave the nest and engage in a wide range of intra- and inter-specific interactions, while queens are typically more sedentary, remain in the safety of the nest, and rarely venture outside. However, in many polygynous ant species including *T. sessile* and *L. humile*, queens frequently travel on trails alongside workers and occasionally engage in independent foraging. Therefore, queens may engage in interactions with unrelated colonies and may be an important factor in mediating interactions between mutually aggressive colonies, including potential colony mergers. The current results advocate for the use of queens in aggression assays as worker-queen and queen-queen aggression assays may be important for assessing the potential mergers between mutually aggressive colonies.

## Methods

### Collection sites

All *T. sessile* colonies were collected across different locations within 80 km radius of Purdue University campus, Tippecanoe County, Indiana (40°25′26.40″N, − 86°55′44.40″W). All colonies were separated by at least 4 km to limit the possibility they were nests of the same colony. To compare nestmate recognition in natural versus urban colonies, 10 colonies were collected in urban areas and 10 in natural areas^9,13^. Urban areas included suburban residential areas with single-family housing and commercial office and retail areas throughout Tippecanoe County. All colonies collected in urban areas were fragments of larger polydomous and polygynous supercolonies and contained a few thousand workers, few hundred queens, and numerous brood. Natural areas were large tracts of mixed hardwood forest that contained mature trees and were free of any anthropogenic influence or disturbance. All colonies collected in natural areas were small and contained approximately a hundred workers, a single queen, and a small amount of brood. All colonies were brought to the lab and placed in plastic trays coated with Fluon to prevent escapes. Urban colonies were large and consisted of approximately 20,000 workers, > 100 queens, and numerous brood. Natural colonies were small and consisted of approximately a hundred workers, a single queen, and some brood. The colonies were provided with drinking water and artificial nests consisting of Petri dishes filled with moist plaster. The colonies were maintained on a 20% sucrose solution and artificial diet^29^.

### Nestmate recognition assays

The level of aggression between ants from different colonies within habitat type (urban or natural) was tested by using dyad interactions within a neutral arena^17^. To assign colony pairings for aggression testing, each of the 10 colonies was paired up with 3 other colonies using Research Randomizer (https://randomizer.org/). The 3 colonies were selected by randomizing a set of 10 numbers while keeping each number unique. This resulted in a total of 30 colony pairings and assured that each colony was tested an equal number of times. To estimate caste specific differences in aggression levels, aggression was assessed in tests with workers (designated as W) vesus workers from natural habitats, workers vs. workers from urban habitats, queens (designated as Q) versus queens from urban habitats, and workers vs. queens from urban habitats. In contrast to urban colonies which contain multiple queens, natural colonies are typically single queen^13^ which precluded performing replicated trials on queens from natural colonies. As a result, tests involving queens from natural habitats were not performed. Four replications were performed for each colony pairing and each type of test resulting in a total of 360 aggression tests for each habitat type. Randomly selected workers or queens were allowed to walk onto a toothpick and were placed sequentially into a small Petri dish (3 cm diameter). The inner wall of the dish was coated with Fluon™ to prevent escapes. For each replicate, the ants were observed continuously and interactions were scored on a 1–4 scale^30^ [1 = ignore, 2 = avoid, 3 = aggression (lunging, brief bouts of biting and/or pulling), 4 = fighting (prolonged aggression, also abdomen curling to deposit defensive compounds)]. The ants were allowed 10 encounters, with each instance of direct physical contact between the ants regarded as an encounter. For each replicate, the mean score of 10 encounters was used in data analysis^20^. In all assays, individual ants were not tested in more than one trial. Colony pairings exhibiting non-injurious aggression (score of 2 or below) were considered non-aggressive whereas colony pairings exhibiting injurious aggression (score of 3 and above) were considered aggressive.

### Laboratory colony fusion assay

Colony fusion experiments were performed to assess the outcome of intraspecific interactions at the colony level. Six urban colonies were collected, extracted from nesting material and maintained in moist plaster nests. The colonies were fed 20% sugar water and maintained at 82 ± 2°F, 40 ± 10% relative humidity, and 14:10 L:D cycle. Experimental colony fragments consisted of 300 workers, 5 queens, and approximately 1 cm^3^ of brood acclimated for 24 h into 9 cm diameter Petri dishes filled with moist plaster. After acclimation, the colonies were placed inside a large testing arena (90 × 90 × 5 cm high), at opposite corners of the arena, diagonally across the arena and 127 cm apart. An empty nest was placed in the center of the arena, 63.5 cm from either colony. The center nest was kept moist and was covered with an opaque lid, while the side nests were allowed to dry and were covered with transparent lids. Ants are attracted to moist and dark environments and moisture and light gradients were then used to stimulate interactions between the two colonies. Food consisting of 20% sugar water was placed on top of the central nest and was available ad libitum during the test. Five colonies were tested in 6 colony pairings (1–2, 1–3, 2–3, 4–5, 6–4, and 6–5) and 3 replicates were performed for each colony pairing, resulting in a total of 18 replicates or 6 colony fragments for each of the 5 colonies used All replicate tests were performed simultaneously and within 1 week of collecting the colonies to minimize the potential of laboratory rearing on aggressive interactions. Control tests consisted of two colony fragments from the same colony. All queens were marked on the abdomen with a small dot of either white (colony 1) or red (colony 2) acrylic paint (Apple Barrel Colors, Norcross, GA, USA) using a 10/0 spotter brush to determine fusion events. Workers were not marked, but 10 randomly selected workers from the merged colony were used to verify the identity of the surviving ants by re-introducing them into their respective source colonies. Workers that were accepted were scored as belonging to that colony while workers that were attacked were scored as belonging to the opposite colony. For each colony pairing, the total number of dead workers was recorded daily for 7 days. Colony pairs were inspected for fusion and dead queens daily for 7 days. Fusion was defined as the presence of all surviving queens, brood, and the majority (> 90%) of surviving workers in the central nest.

### Statistical analysis

To assess whether there is significant variation in aggressiveness among colonies within habitat type (natural or urban), a linear mixed model that compared urban and natural colonies was used^31^. Each of the 10 urban colonies and each of the 10 natural colonies was mapped to a unique location resulting in 20 unique locations. Between-colony variation for a particular habitat type includes any variation due to location (i.e. colony and location are confounded random effects). Analyses were done separately for worker against worker (WW) and queen against queen (QQ) interactions. Let ${y}_{ijk}$ be the average score over 10 interactions where 1 <  = i, j <  = 10 identify ant colonies and k = 1,2,3,4 identifies the four replicates. The mixed model considered is:

$$ y_{ijk} = { }\mu + C_{i} + C_{j} + e_{ijk} $$

$$ C{ }\sim { }N\left( {0,{ }\sigma_{0}^{2} } \right)\quad {\text{and}}\quad {\text{ e}}\sim { }N\left( {0,{ }\sigma^{2} } \right) $$

here $\mu$ is the overall average score of interactions and the $\mathrm{C}$ represent colony-specific contributions to this score. A less aggressive colony would have a negative $\mathrm{C}$, while a more aggressive colony a positive $\mathrm{C}$. Of interest is an estimate of the overall mean score as well as testing whether there exists colony variability in aggressiveness (i.e., testing ${H}_{0}: {\sigma }_{0}^{2}=0$). This was done using a likelihood ratio test comparing the fit of the model assuming colony-specific random effects to the model that does not include them. The inclusion of two random effects from the same distribution in the model precluded the use of specifying the colonies as random effects. Instead, a marginal mixed model, which required specifying the joint covariance matrix was used^31^. All analyses were performed using SAS^32^.

## Data Availability

The datasets used and/or analyzed during the current study available from the corresponding author on reasonable request.
